# Cancer-associated mutations reveal a novel role for EpCAM as an inhibitor of cathepsin-L and tumor cell invasion

**DOI:** 10.1186/s12885-021-08239-z

**Published:** 2021-05-12

**Authors:** Narendra V. Sankpal, Taylor C. Brown, Timothy P. Fleming, John M. Herndon, Anusha A. Amaravati, Allison N. Loynd, William E. Gillanders

**Affiliations:** 1 Department of Surgery, Washington University School of Medicine, 660 South Euclid Avenue, Campus Box 8109, Saint Louis, MO 63110 USA; 2 Siteman Cancer Center, Washington University School of Medicine, St. Louis, MO USA; 3 Norton Thoracic Institute, St. Joseph’s Hospital and Medical Center, 124 W. Thomas Road, Phoenix, 85013 AZ USA

**Keywords:** Cathepsin-L, EpCAM, Thyroglobulin-type-1 domain, Protease inhibitor, Invasion, TCGA

## Abstract

**Background:**

EpCAM (Epithelial cell adhesion molecule) is often dysregulated in epithelial cancers. Prior studies implicate EpCAM in the regulation of oncogenic signaling pathways and epithelial-to-mesenchymal transition. It was recently demonstrated that EpCAM contains a thyroglobulin type-1 (TY-1) domain. Multiple proteins with TY-1 domains are known to inhibit cathepsin-L (CTSL), a cysteine protease that promotes tumor cell invasion and metastasis. Analysis of human cancer sequencing studies reveals that somatic EpCAM mutations are present in up to 5.1% of tested tumors.

**Methods:**

The Catalogue of Somatic Mutations in Cancer (COSMIC) database was queried to tabulate the position and amino acid changes of cancer associated EpCAM mutations. To determine how EpCAM mutations affect cancer biology we studied C66Y, a damaging TY-1 domain mutation identified in liver cancer, as well as 13 other cancer-associated EpCAM mutations. In vitro and in vivo models were used to determine the effect of wild type (WT) and mutant EpCAM on CTSL activity and invasion. Immunoprecipitation and localization studies tested EpCAM and CTSL protein binding and determined compartmental expression patterns of EpCAM mutants.

**Results:**

We demonstrate that WT EpCAM, but not C66Y EpCAM, inhibits CTSL activity in vitro, and the TY-1 domain of EpCAM is responsible for this inhibition. WT EpCAM, but not C66Y EpCAM, inhibits tumor cell invasion in vitro and lung metastases in vivo. In an extended panel of human cancer cell lines, EpCAM expression is inversely correlated with CTSL activity. Previous studies have demonstrated that EpCAM germline mutations can prevent EpCAM from being expressed at the cell surface. We demonstrate that C66Y and multiple other EpCAM cancer-associated mutations prevent surface expression of EpCAM. Cancer-associated mutations that prevent EpCAM cell surface expression abrogate the ability of EpCAM to inhibit CTSL activity and tumor cell invasion.

**Conclusions:**

These studies reveal a novel role for EpCAM as a CTSL inhibitor, confirm the functional relevance of multiple cancer-associated EpCAM mutations, and suggest a therapeutic vulnerability in cancers harboring EpCAM mutations.

**Supplementary Information:**

The online version contains supplementary material available at 10.1186/s12885-021-08239-z.

## Background

Epithelial cell adhesion molecule (EpCAM) is a transmembrane glycoprotein that is expressed at the basolateral membrane of human epithelial tissues [1]. EpCAM is also highly overexpressed in many human epithelial cancers including colorectal, breast, gastric, prostate, ovarian, and lung cancer [2, 3]. EpCAM was the first human tumor-associated antigen to be identified using monoclonal antibodies [4] and has been the target of monoclonal antibody therapy in colorectal cancer [5, 6]. The monoclonal antibody Catumaxomab, which targets EpCAM and causes an anti-cancer immune response, has been approved for the treatment of malignant ascites in Europe [7]. Despite these developments, further research is needed to improve our understanding of EpCAM’s role in epithelial cancers and its potential as a therapeutic target. Recently, we and others have demonstrated that overexpressed EpCAM modulates oncogenic signaling pathways including ERK [8], NF-κβ [9], AP-1 [10], NF-κβ [9] and Wnt/β-catenin [11–13] pathways. EpCAM expression can differentially regulate oncogenic signaling pathways and invasion depending on the cancer type [14]. These studies suggest that the precise role of EpCAM in cancer biology remains to be elucidated.

**Fig. 1:**
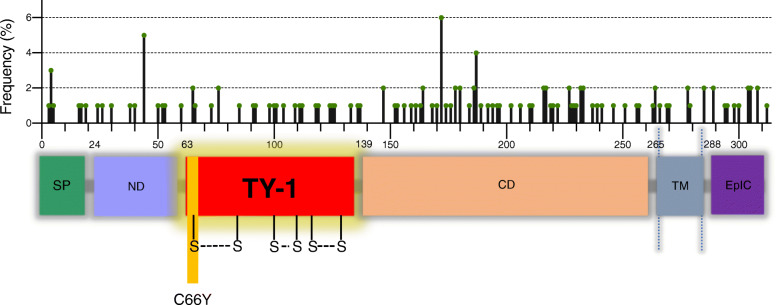
Cancer-associated EpCAM mutations. Histogram (top) shows location and frequency of EpCAM cancer-associated mutations as reported in COSMIC (https://cancer.sanger.ac.uk/cosmic). Protein schematic (bottom) indicates EpCAM protein structure. EpCAM is a type-1 transmembrane protein with a signal peptide (SP, green), an N-terminal domain (ND, blue), a thyroglobulin type-1 domain (TY-1, red), a C domain (CD, tan), a transmembrane domain (TM, gray), and an intracellular domain (EpIC, purple). Domain positions noted as reported by Pavsic et al. [10]. The EpCAM C66Y mutation in the TY-1 domain is predicted to be highly damaging by Polyphen2 (http://genetics.bwh.harvard.edu/pph2/)

At the structural level, EpCAM contains a well characterized thyroglobulin-type-1 (TY-1) domain [15]. The EpCAM TY-1 domain is located between amino acids 63 and 135 in the extracellular region (Fig. 1). There are 17 proteins with TY-1 domains in the human genome (Fig. S1A). TY-1 domains are characterized by a unique sequence motif of 60–80 residues containing six conserved cysteine residues, forming three disulfide bonds (Fig. S1B) [16]. TY-1 domains are conserved in a number of species, and multiple proteins with TY-1 domains function as cathepsin family protease inhibitors [17], (Table S1). Proteins with TY-1 domains have been shown to inhibit cathepsin-L (CTSL), a protease implicated in tumor invasion [18, 19].

CTSL is frequently overexpressed in many cancers, and CTSL expression has been associated with higher histologic tumor grade and metastatic potential [20]. CTSL is secreted by tumor cells [21, 22] and is capable of degrading extracellular matrix proteins and promoting tumor cell invasion [23–25]. Because of its frequent overexpression and prominent role in metastasis, CTSL inhibitors have been the focus of multiple preclinical investigations. CTSL inhibition has demonstrated promising results [20]. Interestingly, both EpCAM [26, 27] and CTSL [28, 29] are known to be secreted into the tumor microenvironment and are present in the serum of human cancer patients. To date, the potential impact of EpCAM on CTSL activity has not been studied.

The Cancer Genome Atlas (TCGA) and other high throughput cancer sequencing studies have dramatically improved our understanding of the genetic basis of cancer. Functional analyses of specific cancer-associated mutations have provided critical insights into how mutated proteins contribute to the biology of cancer, and how to best target these proteins. To date, over 130 coding, cancer-associated mutations in the EpCAM gene have been identified (Fig. 1). Studies reveal that somatic mutations in EpCAM are present in up to 5.1% of some tested cancer cohorts, including squamous and melanoma skin cancers. EpCAM mutations have also been observed in metastatic lesions and other cancers at a frequency of 1–2% (Fig. S1C). No reports to date have investigated the potential role of EpCAM mutations in human cancer, although germline mutations in EpCAM impact cellular localization and are associated with congenital tufting enteropathy and Lynch syndrome [30, 31]. In the current report we demonstrate for the first time that secreted wild type (WT) EpCAM inhibits extracellular CTSL activity. More importantly, we demonstrate that EpCAM cancer-associated mutations alter EpCAM cellular function and localization, abrogate the ability of EpCAM to inhibit CTSL activity, and impact CTSL-driven cancer cell invasion.

## Methods

### EpCAM mutation analysis

To determine the position, amino acid change, and occurrences of somatic EpCAM coding mutations in cancer, we queried the Catalogue of Somatic Mutations in Cancer (COSMIC) publicly available database (https://cancer.sanger.ac.uk/cosmic) at Sanger Institute [32] and tabulated reported mutations. To determine the frequency of EpCAM mutations in tested cancer cohorts, we analyzed 178 non-redundant datasets including 47,005 samples in the cBioPortal for Cancer Genomics [33]. Cell line mutations in EpCAM were analyzed from the Cancer Cell Line Encyclopedia (CCLE) publicly available database at the Broad Institute (https://portals.broadinstitute.org/ccle). The potential impact of mutations of interest on EpCAM protein structure and function was analyzed in silico using PolyPhen-2 [34].

### Cell lines

All cell lines were obtained from the American Type Culture Collection (ATCC, Rockville, MD) and used at a low passage number with the exception of WHIM-3. WHIM-3 is a breast cancer cell line isolated from a patient-derived xenograft. WHIM-3 was provided by the patient derived xenograft (PDX) core at Washington University of School of Medicine (WUSM). All cell lines were maintained in RPMI or DMEM supplemented with 10% FBS and antibiotics (GIBCO BRL, Grand Island, NY). All cell lines were tested for mycoplasma and maintained in ciprofloxacin (10 μg/mL media).

### Recombinant proteins

Recombinant EpCAM-Fc (Gln24-Lys265-IgG1Pro100-Lys300, carrier free) and recombinant CTSL (Glu113-Val333) were purchased from R&D Systems (Minneapolis, MN). Recombinant EpCAM-Fc was reconstituted as recommended by manufacturer and stored at -80 °C in aliquots.

### RNA interference

Lentiviral-mediated RNA interference was performed as previously described [8]. shRNA sequences targeting murine cathepsin-L were provided by the McDonnell Genome Institute at Washington University in St Louis. Specific sequences used for RNA interference are detailed in Table S2.

### Plasmid constructs and site directed mutagenesis

The full-length open reading frame of EpCAM was amplified from the MCF-10A mammary epithelial cell line. The nucleotide sequence was confirmed with NCBI reference sequence NM_002354. The DNA sequence was subcloned into both pcDNA3 (HindIII-XbaI restriction sites) and the retroviral vector pBABE (BamHI-SalI restriction after mutating BamHI sites). EpCAM deletion mutants were generated by PCR amplification and cloned as c-terminal 3X Flag tag fusion protein as shown in Fig. 3e. C66Y EpCAM and other EpCAM cancer-associated mutation constructs were generated using synthetic gene fragments from Integrated DNA Technologies (IDT, Coralville, IA). For example, C66Y EpCAM was generated as a G-block fragment (197G > A; substitution position 197, G➞A). Other mutations were generated based on tumor DNA mutation information available at COSMIC [32] or cBioPortal [33] databases. For EpCAM-GFP fusion constructs generation, EpCAM cDNA was amplified from pCDNA3 vector and subcloned at the N-terminal EGFP of Lentiviral vector pLL3.7 at NheI and AgeI restriction sites.

### Retroviral and Lentiviral transduction

Phoenix-ECO packaging cells were transfected when nearly confluent with 2.5 μg of pBABE-Puro-EpCAM using FuGENE-HD (Promega, Madison, WI). Forty eight hours after transfection viral supernatants were collected, filtered through 0.45 μm filters, and then added to B16-F10, A549 and other cells in media containing 8 μg/mL protamine sulfate. After two successive retroviral infections, cells were grown for 48 h and selected in puromycin for 2 weeks. Lentiviral vector pLL3.7 was obtained from addgene (Plasmid#11795). EpCAM-EGFP fused DNA was transiently transfected in HEK-293 T cells using FuGENE6 or stably transduced as described before [8].

### Flow cytometry

EpCAM expression levels were measured by flow cytometry using PE-labeled EpCAM antibody. Expression was quantified as mean fluorescence intensity (MFI) using a FACScan flow cytometer and FlowJo 10.7.1 software (BD Biosciences, San Jose, CA).

### Cathepsin-L activity assay

Cells were suspended in ice-cold lysis buffer (400 mM sodium phosphate buffer, pH 6.0, 75 mM NaCl, 4 mM EDTA, and 0.25% Triton X-100) and incubated on ice for 30 min. Lysates were centrifuged at 15,000 rpm for 20 min at 4 °C. Protein concentration was determined by the BCA protein assay kit (Pierce, Rockford, IL). CTSL activity was measured by fluorometric assay [35]. Briefly, 10 μg of total cell lysate was diluted in 100 μL of 0.34 M sodium acetate buffer, pH 5.5, containing 2 mM EDTA, and 4 mM dithiothreitol (DTT). To discriminate between CTSL and cathepsin-B activities, a selective cathepsin-B inhibitor, CA074 (Sigma), was added at a final concentration of 5 μM and pre-incubated for 15 min at 37 °C. Fluorogenic substrate Z-Phe-Arg-AMC (Sigma) was added to a final concentration of 5 μM and samples were incubated for an additional 30–60 min at 37 °C. Fluorescence of the degradation product, 7-amino-4-methylcoumarin (7-AMC), was measured at an excitation wavelength of 370 nm and an emission wavelength of 460 nm, using a spectrometer (BioTeK). Cysteine protease inhibitor L-trans-epoxy-succinyl-leucylamido-(4-guanidino)-butane (E64, Sigma) was used as a CTSL inhibitor at 50 μM concentration.

### Protein immunoblots

Cells were washed with ice-cold PBS and lysed in RIPA cell lysis buffer with a protease inhibitor cocktail (Cat#9806, Cell Signaling Technology, Danvers, MA). Protein concentrations were determined by BCA protein assay (Cat#23227, Pierce, Rockford, IL). Approximately 20–30 μg of protein was subjected to SDS-PAGE (NuPAGE, Life Technologies), and transferred by electrophoresis to a PVDF membrane. EpCAM (C-10 cat#25308) and actin-HRP (C4, Cat#sc-47,778) antibodies were obtained from Santa Cruz Biotechnology (Santa Cruz, CA). CTSL antibody (Cat#ab6314) was obtained from AbCAM (Cambridge, MA). Signal detection was performed using the SuperSignal West Pico chemiluminescent immunodetection system (Cat#34580, Thermo Scientific, Rockford, IL). To quantify band density, immunoblots were developed on film, scanned, and pixels in each band were measured using Image J software.

### Immunoprecipitation

For protein extraction, cells were scraped in PBS supplemented with 1 mM Na_3_VO_4_, centrifuged and re-suspended in lysis buffer (20 mM Tris, pH 7.4, 150 mM NaCl, 0.25% NP-40, 0.2% Triton-X, 1 mM Na_3_VO_4_, and 1 mM PMSF). Five hundred μg of protein was precleared with 50 μl Protein A/G-Agarose (sc-2003, Santa Cruz Biotechnology, Santa Cruz, CA) for 30 min. Precleared lysates were then incubated for 1 h using 2 μg of specific antibodies or control IgG at 4 °C with 25 μl of Protein A/G)-Agarose. The immunobeads were washed 3 times in lysis buffer and then eluted in 50 μL of 2× reducing sample buffer, boiled, and proceeded for immunoblotting.

### Immunofluorescence and confocal microscopy

Lentivirus transduced MDCK or transiently transfected HEK-293 T cells with EpCAM-EGFP constructs were lightly trypsinized after 48 h and plated on a glass bottom 35 mm dish with serum-free Opti-MEM media (ThermoFisher Scientific, Grand Island, New York). Cells were visualized and captured with a fluorescence microscope (EVOS digital inverted microscope at 20X or 40X magnifications). Duplicate cell culture was used for data measurements on a Zeiss LSM 880 microscope equipped with the AiryScan detector, an Argon laser (Melles-Griot) for 488 nm excitation and a Zeiss Plan-Apochromat 63 × 1.4 NA DIC M27 Oil objective. The microscope is equipped with temperature and CO_2_ controls that were kept at 37 °C and 5%, respectively.

### EpCAM ELISA

Transiently transfected HEK-293 T or tumor cell lines were plated in 6-well plate overnight and cultured in serum free Opti-MEM media (Cat# 11058021, ThermoFisher Scientific, Grand Island, New York). After 48 h, conditioned media was used as a source of secreted/soluble EpCAM. Human EpCAM DuoSet ELISA kit (Cat#DY960) was used from R&D Systems Inc., Minneapolis, MN as recommended.

### Invasion assays

Cells (4 × 10^4^) were added to Matrigel transwell invasion chambers or control transwell chambers (BD Biosciences, San Jose, CA) and incubated for 24–72 h with chemoattractant media (Clonetics, Walkersville, MD) supplemented with growth factors. Cells invading through the Matrigel or control membranes were fixed using 70% ethanol, stained with 0.1% crystal violet, and photographed in four fields to cover the entire area. Cells were counted from all fields by a scientist blinded to the experimental conditions.

### Animal experiments

Tumor cell expansion for cathepsin-L activity assays were done by xenografting stable lines in 4–6 weeks old female NOD-SCID gamma (NSG) IL2Rgamma^null^ mice (The Jackson Laboratory, Bar Harbor, Maine). Tumor cells were transduced with pBABE-puro retroviruses to express EpCAM or GFP/empty vector as described earlier. Cells were re-suspended in DMEM, 1 × 10^7^ per 100 μL. Before tumor cell injection, the mice were anesthetized with 2.5–4% isoflurane under continuous infusion via a nose mask. Cells were injected subcutaneously then allowed to expand in mice for 2–3 weeks. Two mice were used per cell line to establish cell lines. When the tumor growth reached 0.5–1 cm^3^, tumors were removed aseptically, minced, filtered and plated in appropriate growth media. After 2 days, tumor cells were selected in puromycin containing media for 1 week. These lines were used within 5–8 passages for CTSL activity assay experiments.

### Tumor challenge/lung metastasis assay

Six to ten weeks old female C57BL/6 mice were purchased from Charles River Laboratories (Wilmington, MA) and were used for all lung metastasis experiments. Mice were housed at an institutional animal facility. B16-F10 cells (5 × 10^4^) were re-suspended in 200 μL PBS. For tail vein injections, mice were immobilized in a rodent holder and kept under heating lamp for 1 min to dilate blood vessels. Two hundred μL cell suspensions were injected via tail vein into mice using hypodermic syringes. Animals were monitored weekly. Five mice per group were used in individual studies, and each study was repeated at least three times. Three weeks following tumor challenge, the mice were euthanized by CO_2_ asphyxiation or cervical dislocation according to the approved IACUC protocol. Lung nodules were photographed and counted using a dissecting microscope.

### Statistical analysis

Numerical data are presented as the mean values ± the standard deviation. Statistical significance was evaluated using the Student’s *t* test. A *p*-value < 0.05 was considered to be statistically significant. GraphPad PRISM (GraphPad Software Inc., La Jolla, CA) was used for statistical analysis of all experiments.

## Results

### Somatic EpCAM mutations are present in a significant number of human cancers

To analyze somatic EpCAM mutations in human cancers, we queried the COSMIC database as well as 178 non-redundant datasets including 47,005 samples in the cBioPortal for Cancer Genomics [32, 33]. We identified 115 unique somatic/missense coding EpCAM mutations (Fig. 1). Depending on the dataset and cancer type, EpCAM mutations are present at a frequency between 0 to 5.13% in human cancers (Fig. S1C). Cell line mutations of EpCAM were also analyzed from the CCLE database. We found 33 mutations, including silent, frame shift, and missense mutations (Fig. S1D). We identified multiple cancer-associated mutations which may affect the overall structure and function of EpCAM. C66Y EpCAM is an exemplary TY-1 domain EpCAM cancer-associated mutation identified in a liver cancer specimen. The cysteine residue is part of a critical disulfide bond, and the C66Y mutation is likely to extensively perturb EpCAM structure [15] and thereby disrupt function of the EpCAM TY-1 domain (Fig. 1). Analysis by PolyPhen-2 [34] predicts the C66Y mutation to have a highly damaging effect (data not shown) on protein structure and function. We demonstrate below that the C66Y mutation also impacts EpCAM cellular localization as well.

### WT, but not C66Y EpCAM, inhibits tumor cell invasion in vitro and in vivo

**Fig. 2:**
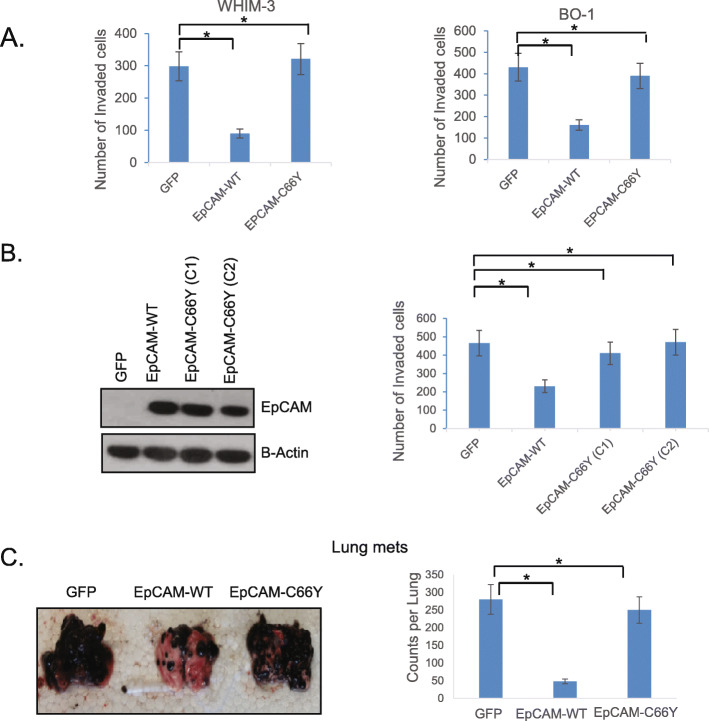
Wild type, but not C66Y EpCAM, inhibits tumor cell invasion in vitro and in vivo. **a** WHIM-3 and PyMT BO-1 breast cancer cell lines were transduced with GFP, wild type EpCAM, or C66Y EpCAM. Invasion was assessed in vitro using Matrigel chambers. **b** B16-F10 cells were transduced with GFP, wild type EpCAM, or C66Y (EpCAM colony 1 and 2). Protein expression is shown by immunoblot (left). Invasion (right) was assessed in vitro using Matrigel chambers. **c** B16-F10 cells were transduced with GFP, wild type EpCAM, or C66Y EpCAM. Transduced cells were injected into animals by tail vein, and the lungs were harvested 2 weeks later. Representative lungs samples are shown (left). Lung metastases were counted using a dissecting microscope by two independent researchers (right). All experiments were performed at least three times. Full-length blots are presented in Supplementary Fig. 5

EpCAM has been implicated in the regulation of cancer invasion. To investigate the role of cancer-associated EpCAM mutations, we initially focused on C66Y. We expressed WT, or C66Y EpCAM, in the human WHIM-3 breast and murine PyMT BO-1 mammary cancer cell lines. Both cell lines have minimal to null endogenous EpCAM expression and an invasive, mesenchymal phenotype with high CTSL activity. Cells were transduced with retroviruses expressing either GFP (control), WT EpCAM, or C66Y EpCAM. Total protein and cell surface expression levels were confirmed by immunoblot and flow cytometry. Total WT and C66Y expression levels were similar, but the C66Y mutant is not expressed on the cell surface (Fig. S2 A-C). Expression of WT EpCAM decreased in vitro invasion in both cell lines approximately 70% compared to cells expressing C66Y EpCAM or GFP (Fig. 2a, b). To extend these findings, we transduced WT or C66Y EpCAM in B16-F10 cells and performed both in vitro and in vivo studies. B16-F10 has minimal to null EpCAM expression (Fig. 2b), is known to be highly invasive, and is dependent on CTSL activity for migration and invasion [36]. Specific ablation of CTSL altered morphology, decreased B16-F10 invasion in vitro, confirming that this cell line is dependent on CTSL for invasion (Fig. S3A-C). We transduced B16-F10 cells with WT or C66Y EpCAM and selected stable clones of the cell lines. We confirmed that expression of WT or C66Y EpCAM was comparable in these cell lines by protein immunoblot (Fig. 2b and S2A, C right panel). Expression of WT, but not C66Y EpCAM, significantly decreased B16-F10 tumor cell invasion in vitro (Fig. 2b, right panel), and the number of lung cancer metastases following tumor challenge in vivo (Fig. 2c, Fig. S3D). CTSL promotes tumor cell invasion and metastasis by degradation of the interstitial matrix and basement membranes. C66Y EpCAM failed to suppress CTSL-mediated lung metastasis in vivo (Fig. 2c). These results suggest that EpCAM expression has the potential to regulate cathepsin-L mediated invasion in the B16-F10 cell line.

### WT, but not C66Y EpCAM, inhibits CTSL activity

**Fig. 3:**
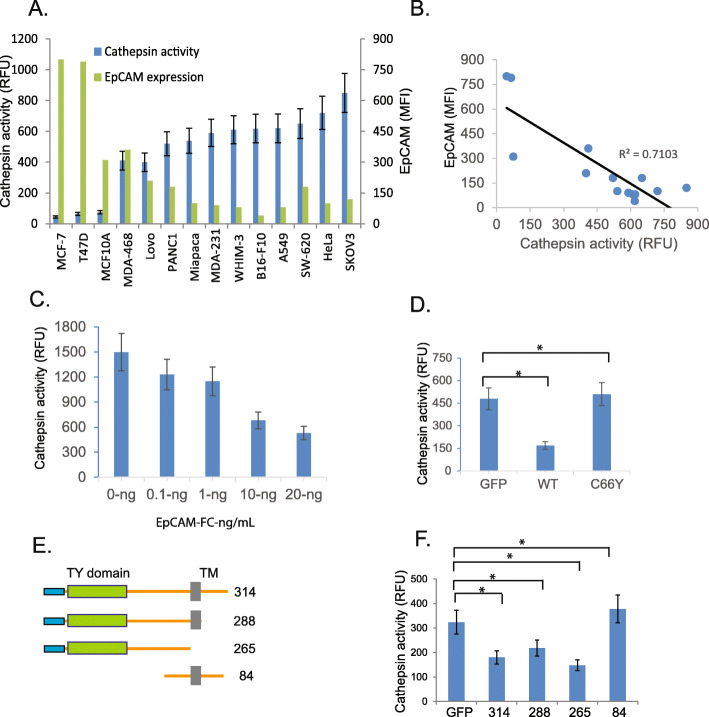
Wild type, but not C66Y EpCAM, can inhibit CTSL activity. **a**, **b** EpCAM expression negatively correlates with CTSL activity in a panel of cancer cell lines. EpCAM expression was measured by flow cytometry, and CTSL activity was measured using a fluorescent substrate. **c** Recombinant EpCAM inhibits CTSL activity. Increasing doses of recombinant EpCAM were added to conditioned media from SKOV-3 cells (source of CTSL). CTSL activity was measured after 30 min. **d** Wild type, but not C66Y EpCAM, inhibits CTSL activity. A549 cells were stably transduced with GFP, wild type EpCAM or C66Y EpCAM. Endogenous CTSL activity was measured. **e**, **f** Wild type EpCAM and EpCAM deletion mutants containing the TY-1 domain, can inhibit CTSL activity. A549 cells were stably transduced with the indicated EpCAM deletion mutants, and endogenous CTSL activity was measured

Based on the known role of TY-1 domains in the regulation of CTSL (Fig. S1A, B, Table S1), we tested the hypothesis that EpCAM can inhibit CTSL. First, we measured EpCAM expression and CTSL activity in a panel of cell lines. EpCAM expression was assessed by flow cytometry, and CTSL activity was assessed using a fluorescent substrate. We observed a striking inverse correlation between EpCAM expression and CTSL activity (Fig. 3a & b). As previously discussed, EpCAM [26, 27] and CTSL [28, 29] are both secreted into the extracellular space where they likely come in contact in the tumor microenvironment. To determine if soluble EpCAM can inhibit CTSL activity, we incubated recombinant EpCAM-Fc with tumor derived (see methods) SKOV3 cell lysates as a source of CTSL (SKOV3 cells have the highest CTSL activity (Fig. 3a) in the panel of tested cell lines). Recombinant EpCAM-Fc decreased CTSL activity in a dose dependent manner, and at 10 ng/mL, CTSL activity was suppressed approximately 60% (Fig. 3c). We also tested the ability of cancer cell lines transduced with EpCAM to inhibit CTSL. In cell line A549, which has minimal endogenous EpCAM expression (Fig. 3a), WT, but not C66Y EpCAM was able to significantly inhibit CTSL activity (Fig. 3d). To evaluate the potential role of the EpCAM TY-1 domain in the inhibition of CTSL, we generated EpCAM deletion mutants (Fig. 3e). Stably transduced A549 cell lines with EpCAM deletion mutants shows roughly equivalent expression of wildtype EpCAM and EpCAM truncation mutants with C-terminal Flag Tag. The EpCAM 265 truncation mutant, which lacks the transmembrane domain, is secreted into the supernatant (Fig. S4A). Stable A549 cells were further cultured in serum-free Opti-MEM media for 24 h and assayed for CTSL activity. Only EpCAM deletion mutants with intact TY-1 domains (EpCAM 314,288,265) were capable of inhibiting CTSL activity (Fig. 3f). As previously reported [15] and shown here, the cysteine at residue 66 forms a critical disulfide bond in the EpCAM TY-1 domain. The C66Y mutation likely impacts protein structure and CTSL inhibition. Taken together, these studies confirm that EpCAM is capable of inhibiting CTSL activity, presumably via the TY-1 domain.

### WT EpCAM physically interacts with CTSL

**Fig. 4:**
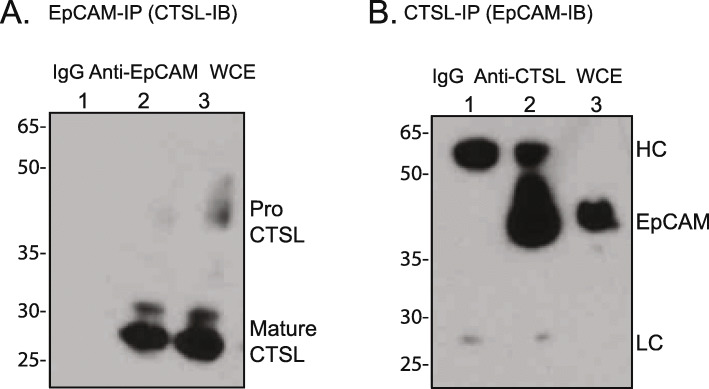
Wild type EpCAM physically interacts with CTSL. **a** MDA-MB-468 cell lysates were immunoprecipitated with EpCAM (clone C-10) or control (IgG) antibody as described in methods section. EpCAM immunoprecipitation pulls down CTSL. **b** Cell lysates were immunoprecipitated with CTSL or control (IgG) antibody. CTSL immunoprecipitation pulls down EpCAM. Full length blots are presented in Supplementary Fig. 5

The ability of TY-1 domain proteins to inhibit CTSL activity typically depends on a physical interaction between the TY-1 domain protein and CTSL. To determine if EpCAM can physically interact with CTSL, we performed immunoprecipitation and protein immunoblot assays. We used the MDA-MB-468 breast cancer cell line, which expresses moderate amounts of both EpCAM and CTSL (Fig. 3a). MDA-MB-468 cell lysates were incubated with IgG, anti-EpCAM, or anti-CTSL antibodies, and immunoprecipitated proteins were immunoblotted for EpCAM or CTSL. EpCAM immunoprecipitation pulls down CTSL (Fig. 4a and Fig. S5), and CTSL immunoprecipitation pulls down EpCAM (Fig. 4b and Fig. S5), demonstrating a physical interaction between these proteins.

### Many cancer-associated mutations prevent EpCAM cell surface expression and secretion and abrogate EpCAM’s ability to inhibit CTSL

EpCAM is a type-I transmembrane glycoprotein, which is predominantly localized on the cell surface. The extracellular domain of EpCAM is secreted (and/or cleaved) and is detectable in cell culture media and the serum and ascites of cancer patients. Soluble/secreted EpCAM (EpEX) is a 242 amino acid (aa) fragment lacking the signal peptide (23 aa), transmembrane domain (23 aa), and cytoplasmic tail (26 aa) [13]. CTSL, while typically expressed in the endosome, has also been shown to be secreted by tumor cells and potentiates invasion in multiple cancer types [37, 38]. A study of congenital tufting enteropathy patients demonstrated that some EpCAM germline mutations can alter cellular trafficking and localization of EpCAM protein to the cell surface [30]. In that study, multiple germline EpCAM mutations prevented EpCAM expression at the cell surface.

To investigate whether cancer-associated EpCAM mutations affect its cellular localization, and/or its potential to inhibit CTSL, we cloned multiple cancer-associated EpCAM mutations in expression vectors alone, or fused to GFP at the C-terminal, and tracked EpCAM localization in vitro. Partial proteolysis of EpCAM is known and is expected with the dominant EpEX protein when transfected in HEK-293 T cells [39]. Immunoblot analysis demonstrates that the majority of EpCAM mutants are expressed at similar levels at WT EpCAM and that the TY-1 domain of EpCAM remains intact when secreted (Fig. S4B & C).

**Fig. 5:**
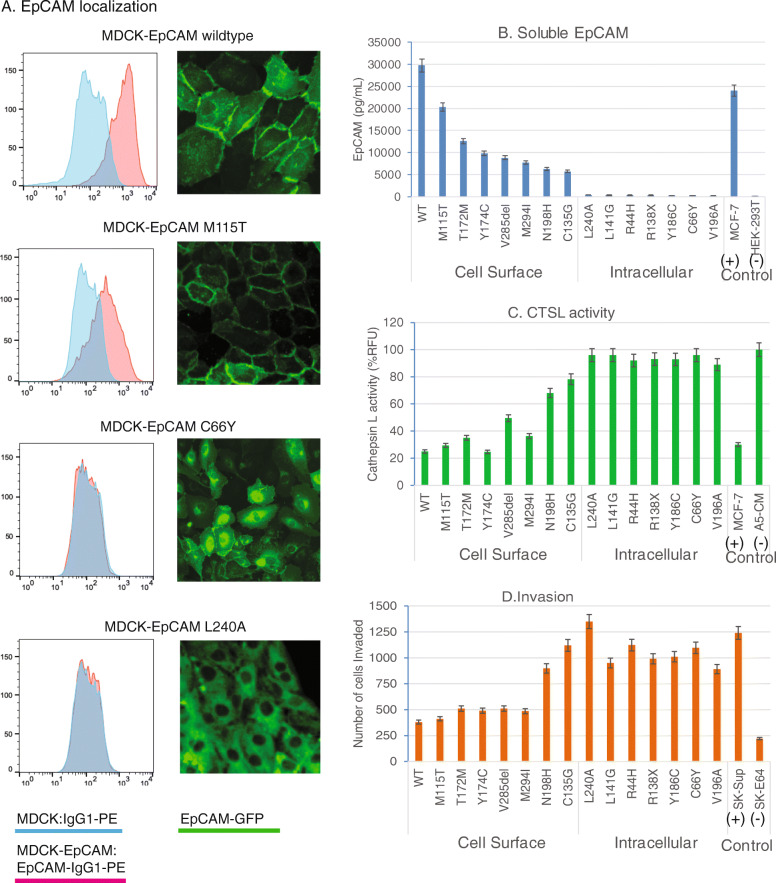
Cancer associated EpCAM mutations prevents EpCAM-CTSL localization, interaction and Cathepsin L mediated invasion. **a** MDCK cells were transduced with WT, M115T, C66Y, and L240A EpCAM and surface expression was analyzed by flow cytometry or confocal microscopy, respectively. **b** GFP-tagged EpCAM mutants were transfected in 12 well culture plates into HEK-293 T cells. 500uL of Opti-mem media was added to harvest secreted EpCAM in conditioned media after 48 h and assayed by ELISA to measure soluble EpCAM. **c** After 48 h of culturing, 1-mL of conditioned media was collected from 6-well SKOV3 culture as a source of soluble CTSL. Conditioned media from transformed HEK-293 T cells was used as a source of soluble EpCAM. CTSL activity was determined as discussed in methods. CTSL activity for secreted/cell surface EpCAM was normalized with EpCAM whole cell extracts blot as shown in supplementary Fig. S4B. A5-CM (A549 conditioned media serves as a control). **d** A549 cells were plated on Matrigel for 6 h. Conditioned media and soluble EpCAM were added, followed by soluble CTSL. After 24 h cells were tabulated to measure invasion

As shown in Fig. 5a, flow cytometry and confocal microscopy demonstrate that representative EpCAM-WT and EpCAM-M115T localize to the cell surface of epithelial MDCK cells, whereas EpCAM-C66Y and EpCAM-L240A localize in the cytosolic compartments. To verify that soluble EpCAM can inhibit CTSL activity, HEK-293 T cells were then transfected with GFP-tagged EpCAM mutants and cultured in serum free media. After 48 h, conditioned media was collected to measure soluble EpCAM levels by ELISA and/or to test the ability of conditioned media to inhibit CTSL activity. As expected, EpCAM mutants that are not expressed at the cell surface were not detected in culture media by ELISA (Fig. 5b, right), and these conditioned medias could not inhibit CTSL activity from SKOV3 media (Fig. 5c, right). In contrast, WT EpCAM and EpCAM mutants expressed at the cell surface were readily detected in culture media (Fig. 5b, left), and conditioned media robustly inhibited CTSL activity (Fig. 5c, left). Together, these results demonstrate that secreted EpCAM inhibits secreted CTSL activity, while cancer-associated EpCAM mutations that prevent cell surface expression also prevent the ability to inhibit CTSL activity.

To confirm and extend these findings, we tested A549 lung cancer cells under multiple conditions. CTSL activity is required for A549 invasion [40] and at baseline A549 cells are less invasive compared to other CTSL-secreting cells. Addition of CTSL-rich SKOV3 conditioned media enhanced A549 invasion 3-fold, and this was abrogated by the CTSL inhibitor E64 (Fig. 5d, last 2 bar graphs). This confirms previous reports that CTSL contributes to A549 invasion. To assess the effect of cancer-associated EpCAM mutations on A549 invasion, A549 cells were plated on Matrigel transwell invasion chambers for 2 h followed by the addition of conditioned media from transfected HEK-293 T cells expressing WT or mutant EpCAM. After 30 min, CTSL-rich SKOV3 conditioned media was added and invasion was monitored over 24 h. As expected, WT and cancer-associated EpCAM mutants expressed at the cell surface suppressed invasion, while cancer-associated EpCAM mutants with no expression at the cell surface did not affect invasion. Together, our findings suggest that soluble/secreted EpCAM can suppress extracellular CTSL protease activity via its TY-1 domain, while cancer-associated EpCAM mutants that are not expressed on the cell surface do not retain this function.

## Discussion

EpCAM is overexpressed in many epithelial cancers, and plays a complex role in cancer biology, stimulating or inhibiting diverse cancer signaling pathways depending on the cancer type [8–10]. There are 17 proteins with TY-1 domains in the human genome (Fig. S1A). TY-1 domains are dependent on three conserved disulfide bonds (Fig. S1B), creating a structure that can function as a cathepsin family protease inhibitor [41, 42]. In studies performed here, we demonstrate that WT EpCAM has the ability to inhibit CTSL, and that this inhibition is dependent on the intact TY-1 domain of EpCAM. C66Y is a cancer-associated EpCAM mutation that disrupts an important disulfide bond in the EpCAM TY-1 domain. The findings reported here provide important insights into the biology of this and other cancer-associated EpCAM mutations.

Cathepsin proteases play a key role in tissue remodeling, tumor invasion, and metastasis. CTSL promotes tumor cell invasion and metastasis by degradation of the interstitial matrix and basement membranes [43]. Cathepsin protease activity is frequently dysregulated during neoplastic transformation, and increased activity within the tumor microenvironment leads to cancer progression, proliferation, metastasis, and drug resistance. CTSL is a powerful lysosomal cysteine proteinase, and CTSL expression and activity is markedly increased in advanced cancers [44]. Forced expression of CTSL is associated with increased migration, invasion, and chemotherapy resistance [36, 45, 46], and inhibition of CTSL activity decreased tumor growth and invasion [47]. As such, a role for WT EpCAM in the regulation of CTSL in normal epithelia and/or cancer (as demonstrated here) is consistent with the known roles for EpCAM in the regulation of both epithelial-to-mesenchymal transition (EMT) and tumor cell invasion.

EpCAM mutations have been identified in many human epithelial cancer types. In the present study we focused on C66Y EpCAM, a mutation resulting in a profound structural and functional alteration in the EpCAM TY-1 domain. We expect other cysteine mutations such as C135G in the TY-1 domain will disrupt EpCAM structure and interaction with CTSL. We also studied 13 other cancer-associated EpCAM mutations and discovered that approximately 50% of these mutations prevented EpCAM cell surface expression. In addition to these nonsynonymous mutations, EpCAM expression is often silenced in cancer cells by promoter methylation, histone modification, and/or aberrant transcription factor signaling [48]. EpCAM is silenced in various cancer types [49, 50], and silencing may exceed the observed rate of mutations. Our data suggest that EpCAM gene silencing is likely associated with increased CTSL activity. Therefore, EpCAM gene silencing may represent an additional therapeutic opportunity to target CTSL, although further studies would be needed.

A previous report showed preferential accumulation of EpCAM C66Y mutant in the endoplasmic reticulum [30]. We show a similar pattern here (Fig. 5a). Unlike C66Y, the L240A mutant does not concentrate in the endoplasmic reticulum (ER), but is expressed diffusely in the cytoplasm (Fig. 5a). It has been suggested that the C66Y mutant may cause aberrant EpCAM dimerization, protein misfolding, and ER retention [30, 51]. A L240A mutation would not affect disulfide bond formation, which is critical to EpCAM structure, and may have minimal impact on protein folding. L240A is located near the transmembrane domain (Fig. 1) and could affect EpCAM membrane anchoring and cell surface expression.

Based on a detailed crystal structure analysis, the first loop of the EpCAM TY-1 domain interacts with the *C*-terminal domain of adjacent EpCAM to form EpCAM *cis*-dimers [15]. This suggests that the EpCAM TY-1 domain may not be available to inhibit CTSL activity when dimerized on the cell surface due to steric hindrances. However, EpCAM is frequently present as a monomer under different experimental conditions [15, 52–54], and EpCAM is known to be cleaved and secreted by cancer cells as shown by us (Fig. 5) and by others [13]. We demonstrate here that soluble EpCAM inhibits CTSL activity in a dose dependent manner (Fig. 3c) and that this inhibitory effect is abrogated by deletion of the TY-1 domain of EpCAM (Fig. 3e). These findings are consistent with other studies demonstrating that thyroglobulin domains in other proteins can inhibit cathepsins (Table S1). Together, these results suggest that EpCAM likely inhibits CTSL in a soluble and/or monomeric state.

These studies reveal a novel role for EpCAM in the regulation of CTSL, but also have potential therapeutic implications as well. Cancers harboring cancer-associated EpCAM mutations and/or EpCAM silencing may have increased CTSL activity. While many cancers demonstrate overexpression of CTSL, it is unclear if this directly corresponds to increased CTSL and metastatic activity. Laboratory techniques have been developed that measure cathepsin activity in tissue specimens, but greater specificity is needed for CTSL [55], and these are not routinely performed. EpCAM mutations could potentially serve as a biomarker of increased CTSL activity in the tumor microenvironment and identify patients that would benefit from protease inhibitors targeting CTSL. Initial clinical trials of protease inhibitors (predominantly matrix metalloprotease inhibitors) showed limited efficacy due to off target activity causing significant drug toxicity [20]. However, more recent preclinical studies have identified and tested multiple different types of cathepsin inhibitors that have been shown to specifically target cathepsin subtypes with less toxicity [56]. Taken together, our findings and these recent preclinical studies suggest that CTSL inhibition in cancers harboring EpCAM mutations may prove to be an effective strategy.

## Conclusions

Our study demonstrates a molecular mechanism of tumor progression in EpCAM mutated tumor cells. CTSL is often overexpressed or activated in advanced epithelial cancers. To date, 115 coding, somatic EpCAM mutations have been reported and deposited in public databases [32, 33]. Our study demonstrates that some of these mutations drive EpCAM accumulation in cytosol compartments and prevent membrane expression. TY-1 domain mutations also disrupted CTSL binding. In an era where genomic testing is being increasingly used to drive biomarker-driven therapeutic decisions in patient care, additional study is required to understand the role of EpCAM mutations in cancer biology, and the potential to target these mutations with existing and/or novel therapies.

## Supplementary Information

**Additional file 1: Supplementary Fig. S1.** A, B. Human proteins with TY-1 domains. A. Seventeen human proteins contain highly conserved TY-1 domains. SMOC-1-2; Testican 1–3; CD74; EpCAM, TACSTD2; IGFBP1–6; and NID1–2. B. Thyroglobulin type-1 domain characterized, alignment of representative TY-1 domain with consensus conserved cysteine residues shown in box. **Supplementary Fig. S1. C**. Cancer-associated EpCAM mutations. Analysis of 178 datasets including 47,005 non-redundant samples from the Cancer Genomics Portal (www.cbioportal.org) reveals cancer-associated EpCAM mutations in approximately 0–5% of analyzed tumor sets. Datasets where the prevalence of cancer-associated mutations are > 1% are shown. **Supplementary Fig. S1. D**. Tested cell lines and EpCAM mutations. The Cancer Cell Line Encyclopedia (CCLE) was queried (https://portals.broadinstitute.org/ccle). A total of 33 mutations in 31 cell lines were identified, including silent, frame shift, and missense mutations. EpCAM mutations were not identified in tested cell lines.

**Additional file 2: Supplementary Fig. S2. A**. Wild type, but not C66Y EpCAM, inhibits tumor cell invasion in vitro and in vivo. WHIM-3 and PyMT BO-1 breast cancer cell lines were transduced with GFP, wild type EpCAM, or C66Y EpCAM. Immunoblot data is shown. B. Wild type, but not C66Y EpCAM, inhibits tumor cell invasion in vitro and in vivo. A, WHIM-3 and B, PyMT BO-1 breast cancer cell lines were transduced with GFP, wild type EpCAM, or C66Y EpCAM. Flow cytometry data is shown.

**Additional file 3: Supplementary Fig. S3.** Specific ablation of CTSL decreases B16-F10 invasion. A-C, To specifically ablate CTSL, B16-F10 cells were stably transduced with a lentivirus expressing shRNAs targeting CTSL and stable clones were selected with variable degrees of CTSL ablation. Specific ablation of CTSL results in (A) altered cell morphology, (B) decreased CTSL activity, and (C) decreased invasion. The CTSL inhibitor E-64 serves as a positive control in the CTSL activity and invasion assays. D, B16-F10 cells stably transduced with GFP or EpCAM were injected into mice by tail vein. Lungs were harvested after 10 days. The number of lung metastases was assessed using a dissecting microscope.

**Additional file 4: Supplementary Fig. S4. A.** EpCAM deletion mutants. (Fig. 3e, f) were expressed at similar levels in A549 cells whole cell extract. EpCAM deletion mutant 265 was secreted in collected media. All constructs were expressed as C-terminal flag tag. Anti-flag antibody was used to develop the immunoblot. EpCAM 291F without signal domain, thus not secreted protein was used as control (not shown in Fig. 3e, f). **Supplementary Fig. S4. B, C**. Immunoblots of tested EpCAM and EpCAM mutants. B. EpCAM-WT and mutants (Fig. 5b) were expressed in HEK-293 T cells. Immunoblot using whole cell extract (WCE) shows surface expressing mutants (left panel) are expressed at similar levels as intracellular mutants (right panel). C. EpCAM mutants expressed as soluble/secreted protein (Fig. 5b, EpEX, 242aa) in cultured HEK-293 T cells were minimally cleaved with an intact TY-1 domain.

**Additional file 5: Supplementary Fig. S5.** Immunoblots of tested EpCAM and EpCAM mutants. A. Full immunoblot scans of EpCAM and EpCAM C66Y expressed in B16-F10 cells (Fig. 2b) and co-immunoprecipitation of EpCAM and CTSL in MDA-MB-468 cells. (Fig. 4a and b) is shown.

**Additional file 6: Table S1.** TY-1 proteins inhibits CTSL family members. Table showing proteins with TY-1 domains have been reported to inhibit cathepsin family with references.

**Additional file 7: Table S2.** Mouse lentiviral CTSL shRNA. Table with lentiviral shRNA sequence used in the current study related to Fig. S3, A-C.

## Data Availability

Human EpCAM mutations were accessed through the publicly available Catalogue of Somatic Mutations in Cancer (COSMIC, https://cancer.sanger.ac.uk/cosmic) at the Sanger Institute. Cell line EpCAM mutations were accessed through the publicly available Cancer Cell Line Encyclopedia (CCLE, https://portals.broadinstitute.org/ccle) at the Broad Institute. The functional impact of the C66Y EpCAM mutation in the TY-1 domain was predicted by Polyphen2 (http://genetics.bwh.harvard.edu/pph2/). The datasets used and/or analyzed during the current study are available from the corresponding author on reasonable request.

## Abbreviations

CTSL: Cathepsin-L
EpCAM: Epithelial Cell Adhesion Molecule
TY-1: Thyroglobulin-type-1 domain
PDX: Patient derived xenograft
