# Supplementary material for: Cancer-associated mutations reveal a novel role for EpCAM as an inhibitor of cathepsin-L and tumor cell invasion
Source: BMC Cancer. 2021 May 12;21:541. doi: 10.1186/s12885-021-08239-z (PMC8114703; doi:10.1186/s12885-021-08239-z)
Supplement: Supplementary file 1 — Additional file 1: Supplementary Fig. S1. A, B. Human proteins with TY-1 domains. A. Seventeen human proteins contain highly conserved TY-1 domains. SMOC-1-2; Testican 1–3; CD74; EpCAM, TACSTD2; IGFBP1–6; and NID1–2. B. Thyroglobulin type-1 domain characterized, alignment of representative TY-1 domain with consensus conserved cysteine residues shown in box. Supplementary Fig. S1. C. Cancer-associated EpCAM mutations. Analysis of 178 datasets including 47,005 non-redundant samples from the Cancer Genomics Portal (www.cbioportal.org) reveals cancer-associated EpCAM mutations in approximately 0–5% of analyzed tumor sets. Datasets where the prevalence of cancer-associated mutations are > 1% are shown. Supplementary Fig. S1. D. Tested cell lines and EpCAM mutations. The Cancer Cell Line Encyclopedia (CCLE) was queried (https://portals.broadinstitute.org/ccle). A total of 33 mutations in 31 cell lines were identified, including silent, frame shift, and missense mutations. EpCAM mutations were not identified in tested cell lines. [file 12885_2021_8239_MOESM1_ESM.pdf]

Fig. S1, A.

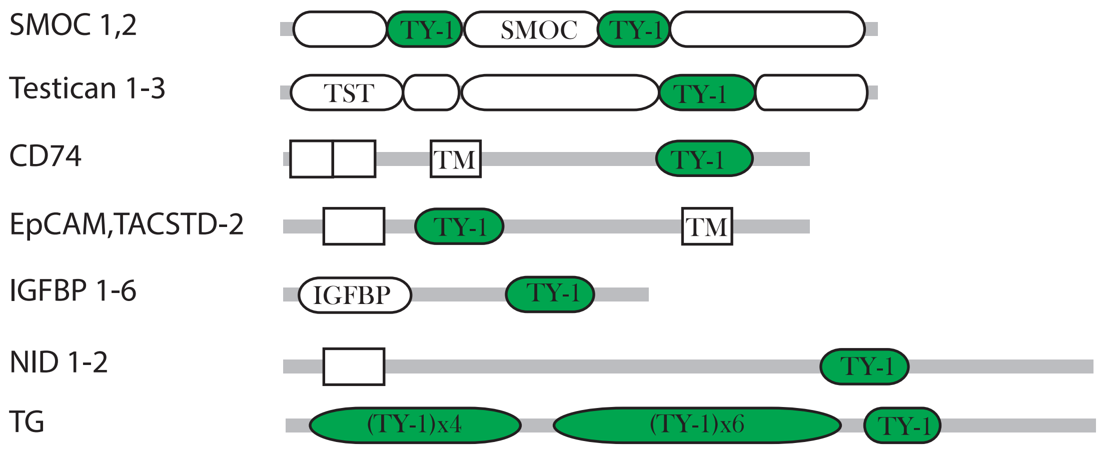

Fig. S1,B.

TG	93-160	LSFCQLQ....YLPQCQDSGDYAPVQC .... DVQHVQCWCVDAE.GMEVYGTRQL.GRPKR.C	NP_003226
SMOC2	213-281	VSSC..ALEZ..AP.C...G....VQC ....GYCWCV...BGBPJGTSRYTPTP....C	NP_001159884
EpCAM	63-135	AAKCLVM....YDPDCDESGLFKAKQC .... NGTSTCWCVNTA.GVRRTDKDTEIT.....C	NP_002345
TACSTD2	70-145	TSKCLLL....YDPDCDPEGRFKARQC ..... NQTSVCWCVNSV.GVRRTDK ...GDLSLRC	NP_002344
IGFBP1	173-251	KEPCRIE....YLPNCNKNGFYHSRQC.......GEAGLCWCVYP..GKRIPGSPEIRGDPN..C	NP_000587
CD74	210-271	LTKCQEE....FRPKCDENGNYLPLQC .... YGSIGYCWCVFPN.GTEVPHTKSR.GRHN..C	NP_001020330
NID1	846-919	KTRCQHE....FVPECDAHGHYAPTQC .... HGSTGYCWCVDRD.GREVEGTRTRPGMTPP.C	NP_002499
Testican1	310-376	GLPCQNE......PRC...G.....QC..........G CWCVD...G....G........AVSC	NP_004589
Consensus		... C.........P.C...G.....QC..........g.CWCVd...…G....g.....g.....C	

WFigure S1,C.

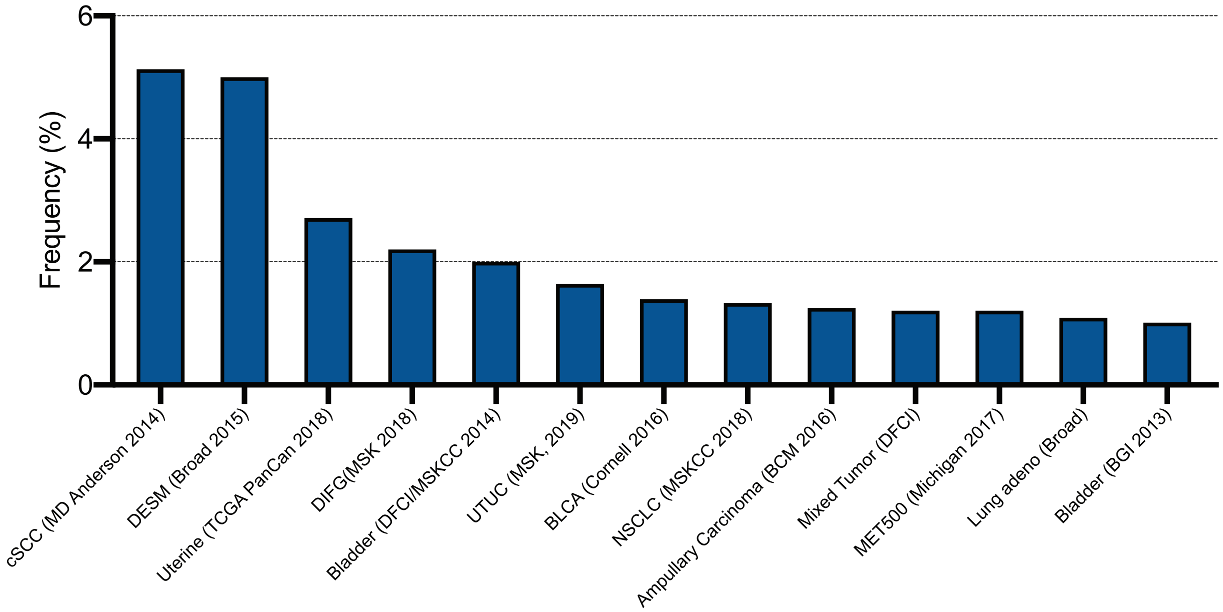
Frequency (%)
6
4
2
0
cSCC (MD Anderson 2014)
DESM (Broad 2015)
Uterine (TCGA PanCan 2018)
DIFG(MSK 2018)
Bladder (DFCI/MSKCC 2014)
UTUC (MSK, 2019)
BLCA (Cornell 2016)
NSCLC (MSKCC 2018)
Ampullary Carcinoma (BCM 2016)
Mixed Tumor (DFCI)
MET500 (Michigan 2017)
Lung adeno (Broad)
Bladder (BGI 2013)

Fig. S1, D.

Hugo Symbol	Entrez Gene Id	Cell line	Variant Classification	Reference	Tumor Seq	Annotation	Protein Change	Amino Acid
EPCAM	4072	HCC38	Silent	C	A	ENST00000263735.4	P3P	3
EPCAM	4072	TOV21G	Frame_Shift	-	C	ENST00000263735.4	P3fs	3
EPCAM	4072	KMS34	Silent	C	T	ENST00000263735.4	L7L	7
EPCAM	4072	LU99	Silent	C	T	ENST00000263735.4	L7L	7
EPCAM	4072	LAN6	Splice_Site	A	G	ENST00000263735.4	E26E	26
EPCAM	4072	MOT	Missense	A	T	ENST00000263735.4	Q45L	45
EPCAM	4072	EB2	Silent	T	C	ENST00000263735.4	N55N	55
EPCAM	4072	MFE319	Missense	A	G	ENST00000263735.4	T56A	56
EPCAM	4072	JHOS2	Missense	C	T	ENST00000263735.4	P84S	84
EPCAM	4072	JEKO1	Missense	C	T	ENST00000263735.4	P84S	84
EPCAM	4072	SKES1	Missense	G	A	ENST00000263735.4	A107T	107
EPCAM	4072	M14	Missense	A	G	ENST00000263735.4	K108R	108
EPCAM	4072	NCIH513	Missense	A	C	ENST00000263735.4	Q109P	109
EPCAM	4072	HCC202	Missensen	C	A	ENST00000263735.4	N111K	111
EPCAM	4072	SNUC5	Missense	A	G	ENST00000263735.4	N120S	120
EPCAM	4072	NCIH1975	Silent	G	A	ENST00000263735.4	Q167Q	167
EPCAM	4072	EFO27	Missense	C	T	ENST00000263735.4	T182M	182
EPCAM	4072	NBSUSSR	Missense	C	T	ENST00000263735.4	T192I	192
EPCAM	4072	RH18	Missense	C	A	ENST00000263735.4	Q201K	201
EPCAM	4072	HEC59	Frame_Shift	-	A	ENST00000263735.4	Q201fs	201
EPCAM	4072	SNGM	Frame_Shift	-	A	ENST00000263735.4	Q201fs	201
EPCAM	4072	MOLM16	Frame_Shift	-	A	ENST00000263735.4	Q201fs	201
EPCAM	4072	HCC2998	Missense	A	C	ENST00000263735.4	Y215S	215
EPCAM	4072	SNU1040	Missense	G	A	ENST00000263735.4	G222S	222
EPCAM	4072	SUIT2	Missense	C	A	ENST00000263735.4	S228Y	228
EPCAM	4072	WM88	Missense	C	A	ENST00000263735.4	S228Y	228
EPCAM	4072	HS840T	Silent	A	G	ENST00000263735.4	Q239Q	239
EPCAM	4072	2313287	Silent	T	C	ENST00000263735.4	V252V	252
EPCAM	4072	CORL303	Silent	T	C	ENST00000263735.4	D253D	253
EPCAM	4072	CORL303	Missense	A	T	ENST00000263735.4	Q262L	262
EPCAM	4072	NCIH1734	Missense	G	T	ENST00000263735.4	A278S	278
EPCAM	4072	NCIH1792	Missense	G	T	ENST00000263735.4	V285L	285
EPCAM	4072	HPBALL	Missense	C	T	ENST00000263735.4	A314V	314
