# Supplementary material for: Cancer-associated mutations reveal a novel role for EpCAM as an inhibitor of cathepsin-L and tumor cell invasion
Source: BMC Cancer. 2021 May 12;21:541. doi: 10.1186/s12885-021-08239-z (PMC8114703; doi:10.1186/s12885-021-08239-z)

Fig. S2.

A.

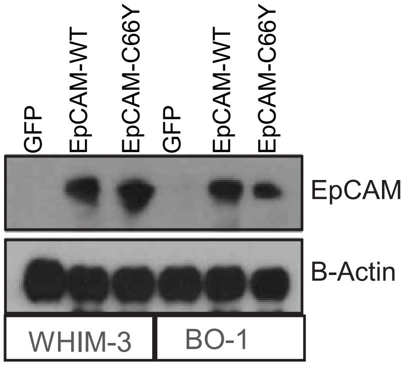

Figure S2A. Wild type, but not C66Y EpCAM, inhibits tumor cell invasion in vitro and in vivo. WHIM-3 and PyMT BO-1 breast cancer cell lines were transduced with GFP, wild type EpCAM, or C66Y EpCAM. Immunoblot data is shown.

B. WHIM-3: EpCAM flow cytometry

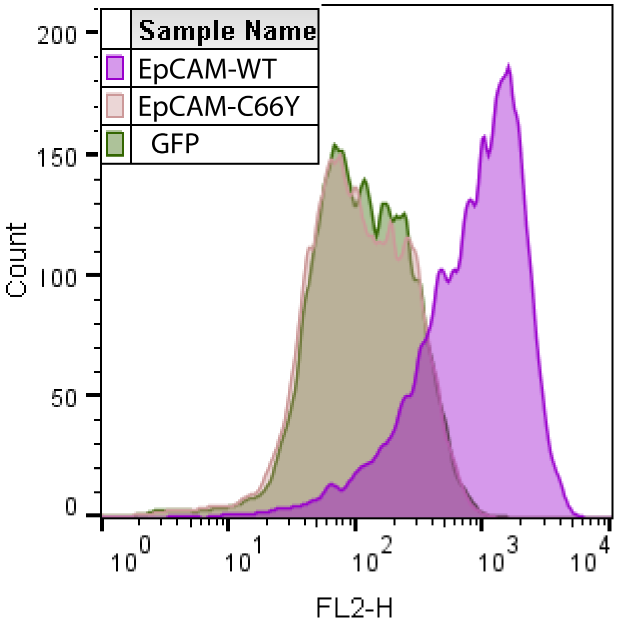

C. BO-1: EpCAM flow cytometry

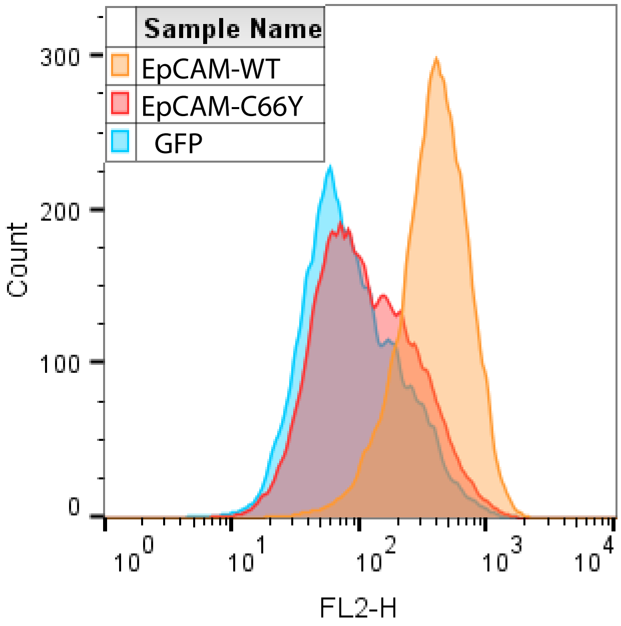

Supplement: Supplementary file 2 — Additional file 2: Supplementary Fig. S2. A. Wild type, but not C66Y EpCAM, inhibits tumor cell invasion in vitro and in vivo. WHIM-3 and PyMT BO-1 breast cancer cell lines were transduced with GFP, wild type EpCAM, or C66Y EpCAM. Immunoblot data is shown. B. Wild type, but not C66Y EpCAM, inhibits tumor cell invasion in vitro and in vivo. A, WHIM-3 and B, PyMT BO-1 breast cancer cell lines were transduced with GFP, wild type EpCAM, or C66Y EpCAM. Flow cytometry data is shown. [file 12885_2021_8239_MOESM2_ESM.pdf]
