# Supplementary figures and images for: Cancer-associated mutations reveal a novel role for EpCAM as an inhibitor of cathepsin-L and tumor cell invasion
Source: BMC Cancer. 2021 May 12;21:541. doi: 10.1186/s12885-021-08239-z (PMC8114703; doi:10.1186/s12885-021-08239-z)

Fig. S3.

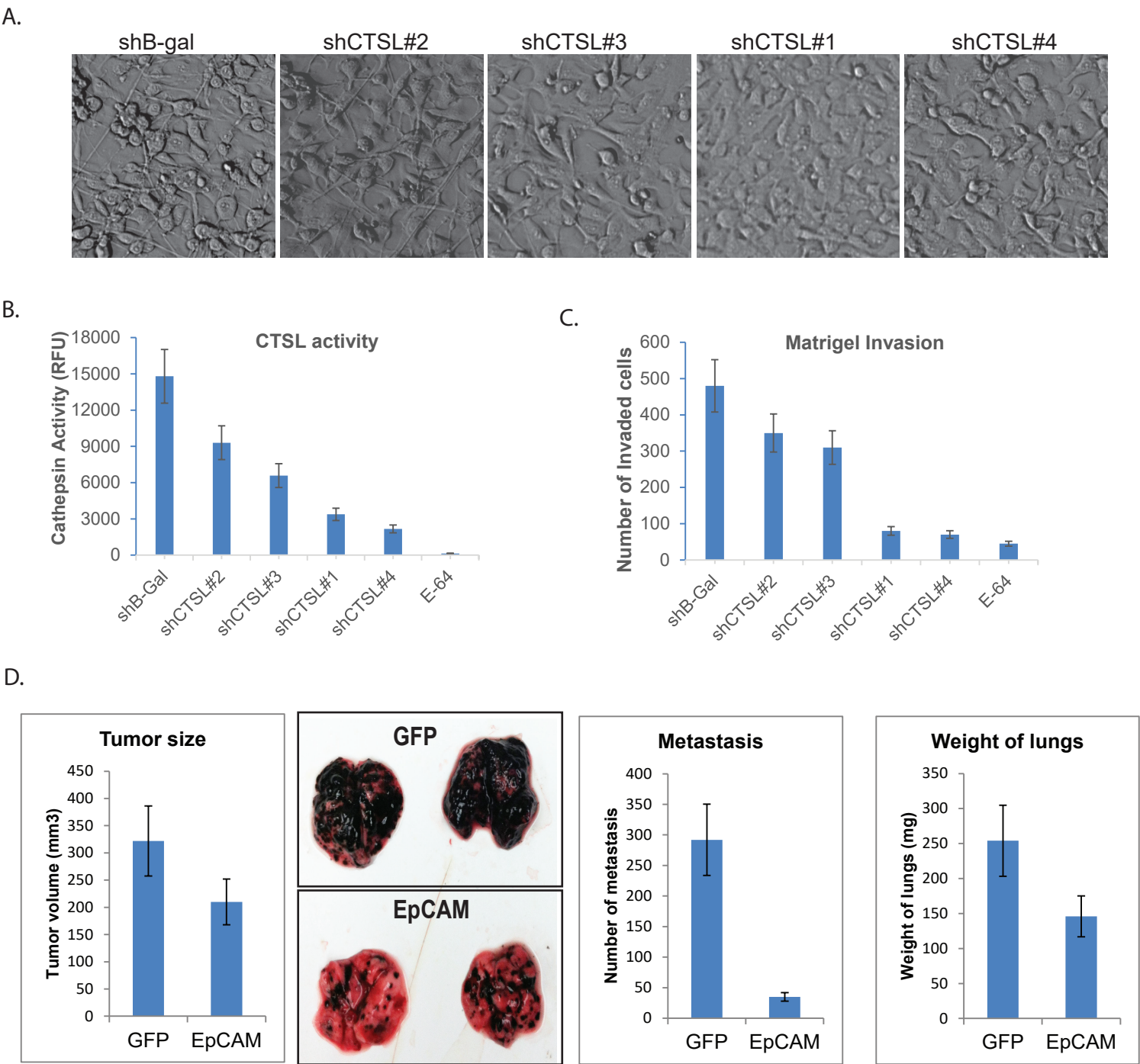

Supplement: Supplementary file 3 — Additional file 3: Supplementary Fig. S3. Specific ablation of CTSL decreases B16-F10 invasion. A-C, To specifically ablate CTSL, B16-F10 cells were stably transduced with a lentivirus expressing shRNAs targeting CTSL and stable clones were selected with variable degrees of CTSL ablation. Specific ablation of CTSL results in (A) altered cell morphology, (B) decreased CTSL activity, and (C) decreased invasion. The CTSL inhibitor E-64 serves as a positive control in the CTSL activity and invasion assays. D, B16-F10 cells stably transduced with GFP or EpCAM were injected into mice by tail vein. Lungs were harvested after 10 days. The number of lung metastases was assessed using a dissecting microscope. [file 12885_2021_8239_MOESM3_ESM.pdf]

Fig. S4:

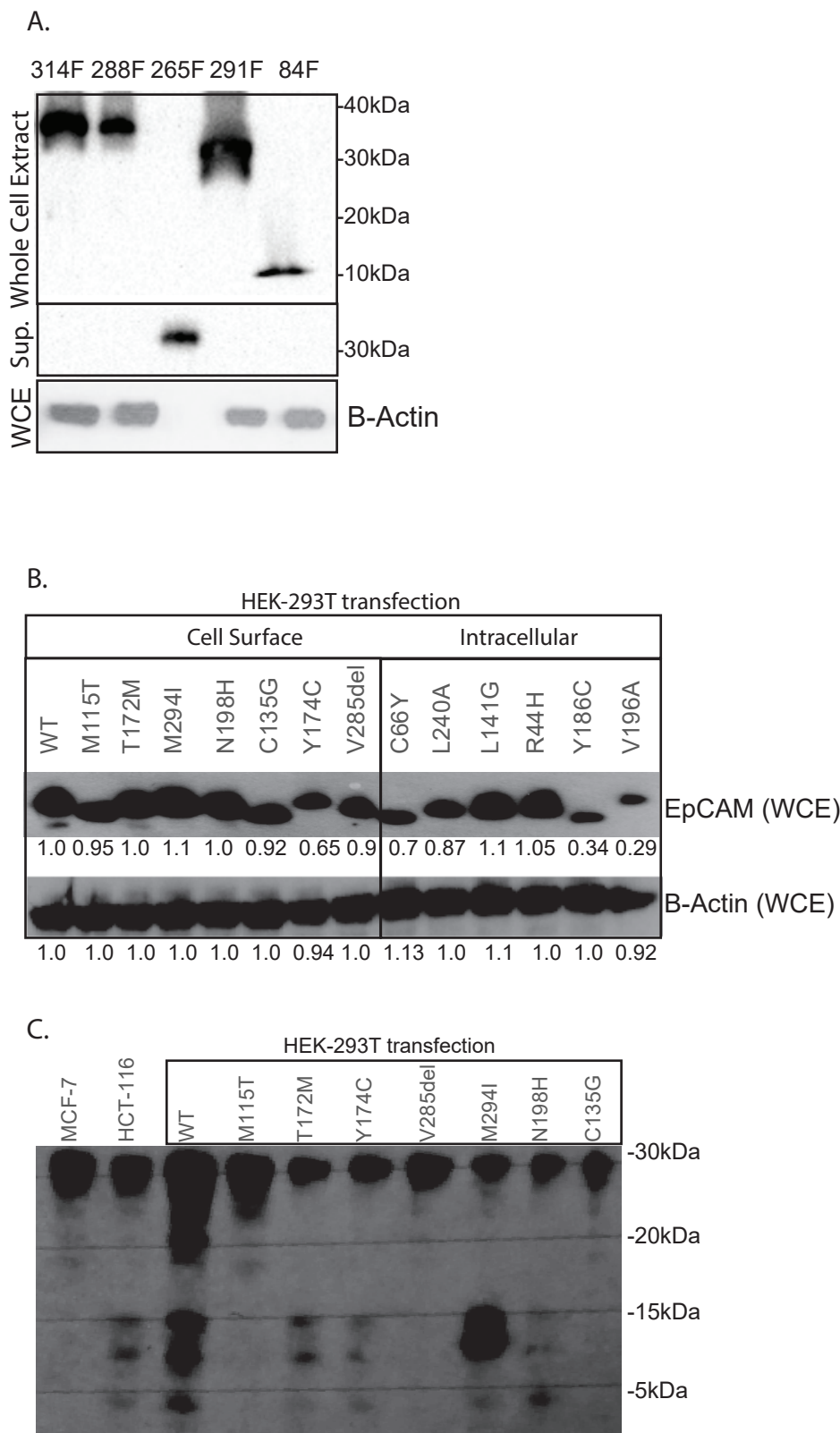

Supplement: Supplementary file 4 — Additional file 4: Supplementary Fig. S4. A. EpCAM deletion mutants. (Fig. 3e, f) were expressed at similar levels in A549 cells whole cell extract. EpCAM deletion mutant 265 was secreted in collected media. All constructs were expressed as C-terminal flag tag. Anti-flag antibody was used to develop the immunoblot. EpCAM 291F without signal domain, thus not secreted protein was used as control (not shown in Fig. 3e, f). Supplementary Fig. S4. B, C. Immunoblots of tested EpCAM and EpCAM mutants. B. EpCAM-WT and mutants (Fig. 5b) were expressed in HEK-293 T cells. Immunoblot using whole cell extract (WCE) shows surface expressing mutants (left panel) are expressed at similar levels as intracellular mutants (right panel). C. EpCAM mutants expressed as soluble/secreted protein (Fig. 5b, EpEX, 242aa) in cultured HEK-293 T cells were minimally cleaved with an intact TY-1 domain. [file 12885_2021_8239_MOESM4_ESM.pdf]

Fig. S5:

Figure 2B.

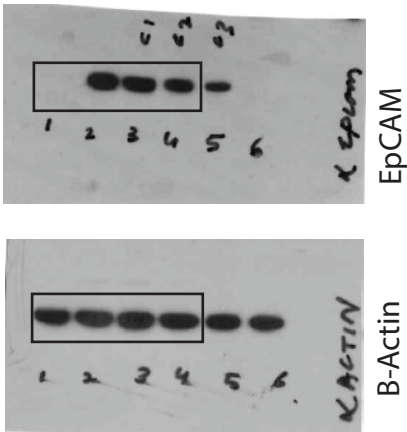

Figure 4A.

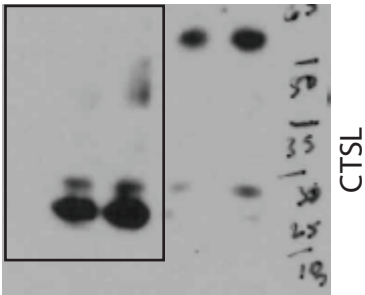

Figure 4B.

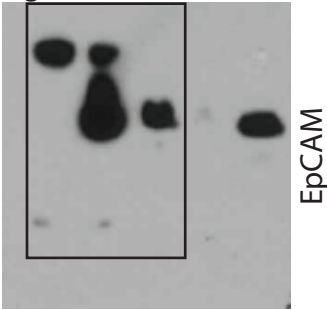

Supplement: Supplementary file 5 — Additional file 5: Supplementary Fig. S5. Immunoblots of tested EpCAM and EpCAM mutants. A. Full immunoblot scans of EpCAM and EpCAM C66Y expressed in B16-F10 cells (Fig. 2b) and co-immunoprecipitation of EpCAM and CTSL in MDA-MB-468 cells. (Fig. 4a and b) is shown. [file 12885_2021_8239_MOESM5_ESM.pdf]
