# Supplementary material for: Cancer-associated mutations reveal a novel role for EpCAM as an inhibitor of cathepsin-L and tumor cell invasion
Source: BMC Cancer. 2021 May 12;21:541. doi: 10.1186/s12885-021-08239-z (PMC8114703; doi:10.1186/s12885-021-08239-z)
Supplement: Supplementary file 6 — Additional file 6: Table S1. TY-1 proteins inhibits CTSL family members. Table showing proteins with TY-1 domains have been reported to inhibit cathepsin family with references. [file 12885_2021_8239_MOESM6_ESM.pdf]

Supplementary Table S1. TY-1 proteins inhibits CTSL family members.

TY-1 domain proteins	Protease inhibited	References
Testican	cathepsin L	1,2
Equistatin	cathepsin B, D	3,4
Saxiphilin	cathepsin L	5
Thyroglobulin	cathepsin L	6
p41 fragment	cathepsin L	7
IGFBPs	cathepsin L	8
NID	cathepsin L, K	9

References

1. Bocock, J.P., Edgell, C.J., Marr, H.S., and Erickson, A.H. (2003). Human proteoglycan testican-1 inhibits the lysosomal cysteine protease cathepsin L. Eur. J. Biochem. 270, 4008–4015.
2. Meh, P., Pavsic, M., Turk, V., Baici, A., and Lenarcic, B. (2005). Dual concentration-dependent activity of thyroglobulin type-1 domain of testican: specific inhibitor and substrate of cathepsin L. Biol. Chem. 386, 75–83.
3. Lenarcic, B. and Turk, V. (1999). Thyroglobulin type-1 domains in equistatin inhibit both papain-like cysteine proteinases and cathepsin D. J. Biol. Chem. 274, 563–566.
4. Lenarcic, B., Ritonja, A., Strukelj, B., Turk, B., and Turk, V. (1997). Equistatin, a new inhibitor of cysteine proteinases from Actinia equina, is structurally related to thyroglobulin type-1 domain. J. Biol. Chem. 272, 13899–13903.
5. Lenarcic, B., Krishnan, G., Borukhovich, R., Ruck, B., Turk, V., and Moczydlowski, E. (2000). Saxiphilin, a saxitoxin-binding protein with two thyroglobulin type 1 domains, is an inhibitor of papain-like cysteine proteinases. J. Biol. Chem. 275, 15572–15577.
6. Pungercic, G., Dolenc, I., Dolinar, M., Bevec, T., Jenko, S., Kolaric, S., and Turk, V. (2002). Individual recombinant thyroglobulin type-1 domains are substrates for lysosomal cysteine proteinases. Biol. Chem. 383, 1809–1812.
7. Guncar, G., Pungercic, G., Klemencic, I., Turk, V., and Turk, D. (1999). Crystal structure of MHC class II-associated p41 Ii fragment bound to cathepsin L reveals the structural basis for differentiation between cathepsins L and S. EMBO J. 18, 793–803.
8. Fowlkes, J.L., Thrailkill, K.M., George-Nascimento, C., Rosenberg, C.K., and Serra, D.M. (1997). Heparin-binding, highly basic regions within the thyroglobulin type-1 repeat of insulin-like growth factor (IGF)-binding proteins (IGFBPs) -3, -5, and -6 inhibit IGFBP-4 degradation. Endocrinology 138, 2280–2285.
9. Primož Meh; Miha Pavšič; Vito Turk; Brigita Lenarčič; et al. Thyroglobulin type-1 domain of nidogen inhibits cathepsin L and cathepsin K. International Conference on Cysteine Proteinases and Their Inhibitors. 2002. ISBN:9616303384.
