# Supplementary material for: Cancer-associated mutations reveal a novel role for EpCAM as an inhibitor of cathepsin-L and tumor cell invasion
Source: BMC Cancer. 2021 May 12;21:541. doi: 10.1186/s12885-021-08239-z (PMC8114703; doi:10.1186/s12885-021-08239-z)
Supplement: Supplementary file 7 — Additional file 7: Table S2. Mouse lentiviral CTSL shRNA. Table with lentiviral shRNA sequence used in the current study related to Fig. S3, A-C. [file 12885_2021_8239_MOESM7_ESM.pdf]

Supplementary Table S2. Mouse lentiviral CTSL shRNA

**Clone Name**	**Sequence**	**shRNA #**
NM_009984.2-1066s1c1	CCAGCTATCCTGTCGTGAATT	shCTSL#1
NM_009984.2-639s1c1	GCTTTCCAGTACATTAAGGAA	shCTSL#2
NM_009984.2-851s1c1	CCAGTTCTATAGTTCAGGCAT	shCTSL#3
NM_009984.2-191s1c1	CAGAAGACTGTATGGCACGAA	shCTSL#4
